# A Clinical Report of Chest Affection

**Published:** 1828-04

**Authors:** Powell Charles Blackett

**Affiliations:** Member of the Royal College of Surgeons, London; Surgeon Royal Navy; and Surgeon Extraordinary to his Royal Highness the Duke of Clarence, &c. &c.


### AFFECTION OF THE CHEST.

*A Clinical Report of Chest Affection.* By POWELL CHARLES BLACKETT, Member of the Royal College of Surgeons, London; Surgeon Royal Navy; and Surgeon Extraordinary to his Royal Highness the Duke of Clarence, &c. &c.

F. H., ætatis sixty-five, by trade a plasterer, sent for me on the 17th of December last. He complained of great exhaustion, from having, during the night, coughed up a considerable quantity of blood. This patient had been a hard drinker for more than twenty years, and had laboured under a severe cough during the last two, the remains of an attack of pneumonia, expectorating daily from eight to nine fluid ounces of mucus, and that too occasionally streaked with blood; complaining always of pain in the left side, and great difficulty of breathing, especially in the horizontal position; with frequent attacks of vertigo, seizing him often in the streets so suddenly as to compel him to hold by the rails for support. He had considerable pain about the sternal extremity of the eighth rib of the left side, which darted occasionally to the same extremities of the seventh and ninth, and then, leaving these anterior parts, would fix itself suddenly in the dorsal extremities of the three last-mentioned ribs. Always, since the attack of pneumonia, he has experienced a difficulty in ascending a ladder or steep staircase, his breath stopping, attended with a complete constriction about the lower end of the sternum, with occasional pain at the scrobiculus cordis. His bowels are always in a state of constipation, yet the motions are of a healthy colour. He is frequently passing urine, which is of a higher colour than usual. Whenever the phlegm accumulates, he is seized with vertigo, continuing until the cough comes on: this, which is spasmodic, never ceases until the fluid is fully expectorated, when he finds relief, and falls into a sound sleep. These attacks are more frequent during the night than in the day. He can lie at times on either side or back, but frequently he can do neither, but is obliged to be propped up. He is much influenced by the weather, especially by changes from a mild atmosphere to fog or frost, and by the east wind: the consequences resulting from this alteration in the state of the weather are ever fresh excitements to his various symptoms, rendering his life an accumulated state of disease and wretchedness. His pulse, generally speaking, is frequent and weak; his thirst at times excessive; his appetite bad; his appearance cachectic; his body much emaciated; and his whole frame, indeed, so exceedingly irritable, that the slightest cause will excite an universal tremor and agitation, followed by violent coughing, &c.

December 17th.—I found my patient much exhausted from the large quantity of blood he had coughed up, which was full twelve fluid ounces, florid and coagulated. He said he had been unwell for the last ten days or fortnight, but that yesterday afternoon he was taken suddenly ill, with shivering, accompanied with great difficulty of breathing and continued cough; that, between the hours of nine and ten, these symptoms increased, attended by great pain about the eighth rib on both sides, and slight pain at the scrobiculus cordis; that he expectorated much phlegm streaked with florid blood, and then fell asleep, and rested tolerably well until between three and four o'clock this morning, when every symptom was so aggravated that he started suddenly from his sleep, with a strong feeling of suffocation, utterly unable to remain in the horizontal posture, finding no relief except in sitting up and throwing himself forward. This orthopnea lasted about fifteen minutes, when the cough commenced, and he threw up the blood before mentioned. After this discharge, he found himself greatly relieved, and could slightly recline on his back; but neither cough nor expectoration left him, he continuing to throw up much viscid phlegm and coagulated blood. It was five o'clock P.M. before I was sent for to attend him, and consequently did not see him until the above symptoms had taken place. His attendants told me he had been fully employed in his business, and exposed to the weather, for the last two or three months, and that he appeared much as usual until the 7th of December, when the south-west winds oppressed and relaxed him; and that, on the

15th, the wind becoming due east, with foggy weather, he began to complain of great difficulty of breathing, pain, &c.

The quantity of mucus and coagulated blood, independently of the discharge of pure blood during the night, was about sixteen fluid ounces. The viscidity of the phlegm was so great, that it required great agitation to separate it from the sides of the vessel: water was added, and it floated, but the coagulated blood sunk to the bottom; the phlegm was transparent and colourless. The respiration was tracheal, with the mucous wheeze; his voice vibrated, and was louder than natural, and, strange to say, he did not stutter, although, when in a state of health, and indeed to the present time, he could not articulate half a dozen words without most considerable stammering! His pulse was quick, small, and wiry: he had great pain in the head, pallid countenance, dry tongue and foul, parched skin, great thirst, and confined bowels; he passed little or no urine, and had a fixed pain distressing him about the scrobiculus cordis, as well as at the sternal extremities of the six inferior ribs on both sides.

In this case I did not deem blood-letting expedient, owing to the debilitated state of my patient, in whom I am satisfied it could answer no good purpose, but, on the contrary, would have weakened him, and deprived him of that strength so necessary for combating the disease. We must be all aware that much mischief has been done by the injudicious and undistinguished use of the lancet. Armstrong says, in his Medical Essays, anno 1773, "The first thing that is commonly done in a fever is to let blood: if the patient is nothing better next day, if even worse, perhaps for his loss of blood the day before, the bleeding is repeated, *because* it is a fever;" and he further adds, "I am positive it is talking very much within bounds to say, that many more Englishmen die by the lancet at home than by the sword abroad." My patient had likewise excessive impatience and irritability, accompanied by an exceeding dread of the lancet. I therefore pursued the following plan of emptying the bowels, allaying excitement, and causing counter-irritation.

> ℞. Hydr. Submur. gr. iij.; Extr. Colocynth. C. gr. vj. fiant pilulæ duæ statim deglut.—℞. Magnes. Sulph. ℥ss.; Infus. Sennæ ℥j.; Aq. Menth. Pip. ʒss. M. fiat haust. post horam unam sumendus.—Empl. Lytta sterno applicandum.—℞. Potass. Acetat. ʒj.; Syrup. Papav. ℥ss.; Aq. Menth. Virid. ℥vss. M. capiat quartam partem quartâ quâque horâ.—Diet: whey, toast-and-water, and fruit.

18th.—Respiration rather easier; could lie completely on his back; still the mucous wheeze. Bowels have been opened several times, with not the least appearance of blood in the discharge, but accompanied profusely by mucus. Pain at the scrobiculus cordis less; pain in the chest less on the right side—on the left as before; pulse as yesterday; tongue clean, skin moist. Has voided his urine five times, about three ounces at each time: it is high coloured, with the brickdust sediment; pain of the head less, but feels very giddy when he moves. Between the hours of three and four, he had another attack of orthopnea, after which he threw up about seven ounces of blood mixed with phlegm.

Medicamenta repet$^{r}$, sine pilulis.

19th.—As yesterday.

20th.—Somewhat easier; but seized, as usual, between three and four in the morning; the quantity of blood thrown up was only three ounces. Phlegm very viscid, and of a yellow colour; pulse much the same; urine less in quantity, yet of a high colour, with the same sediment. Was in a profuse state of perspiration for two hours. Bowels confined.

Ordered to continue his mixture and diet, with the following—℞. Ext. Colocynth. C. gr. x.; Hyd. Submur. gr. iv. fiant pil. iij. h. s. sumendæ.

21st.—Respiration easy. Bowels opened five times, not any appearance of blood in the evacuations. Pulse soft and regular, about ninety; skin moist; was in a high state of perspiration this morning for three hours; urine the same in quantity and appearance. Had an attack during the night as usual, but expectorated only two ounces of blood. The quantity and colour of the phlegm as yesterday.

Mist. repet$^{r}$, cum eadem diæta.

22d.—He is today much worse than yesterday, complaining of great difficulty of breathing, so much so, that to breathe at all he is obliged to be propped up in bed. Bowels once opened. Has passed very little urine, and that of a dark red colour, without sediment. Has expectorated more phlegm than yesterday, and it is more streaked with blood, and the colour is darker. Was in a profuse perspiration during the night, which commenced as soon as the usual attack subsided. My patient not being so irritable today, I got him to consent to the application of the stethoscope. Having removed his flannel shirt, &c. I applied the instrument, without the stopper, to the sternal extremity of the ninth rib on the left side, where the respiratory murmur was absent. I then applied it to the seventh rib, from its dorsal to its sternal extremity, with the same issue. The third application was to the fifth and sixth ribs, from one extremity to the other, where I perceived the mucous wheeze, showing that there was a copious discharge, or rather extravasation, into the air-cells and bronchial tubes. I then removed the instrument, and applied it to the axilla, where the mucous wheeze was yet stronger, causing to the ear a sensation like gurgling. On the right side, the stethoscope was applied as on the left: the respiration gave the mucous wheeze, unattended by the gurgling in the axilla. On percussion, the sound was dull in every part of the chest, excepting the axilla on the right side. He complains today of great pain at the sternal extremities of the seventh, eighth, and ninth ribs of the left side, and at the scrobiculus cordis, attended with excessive nausea; his pulse is quick and full, about 112; formerly his pulse was always quick, weak, or small. I waited twenty minutes, and again tried the pulse: it was then as usual, quick, weak, and small. This state of the pulse again did away with all idea of blood-letting. PERCIVAL says, in his Essays, "But when the disease is of a mixed kind,—when the lungs are not so much inflamed, or loaded with a pituitous matter,—when bleeding gives but little relief,—when the pulse, though quick, is small,—when the patient is not able to bear evacuations, and the disease hath continued for some time, in such circumstances epispastics will produce remarkably good effects." Upon this doctrine, I ordered a large blister to the lower extremity of the sternum, with five grains of Calomel going to bed, and the following mixture:

℞. Magnes. Sulph. ʒiij.; Acid. Sulph. dilut. m. xl.; Syr. Papav. ℥ss.; Aq. puræ ℥vss. M. fiat mist. cujus capiat coch. iij. sextâ quâque horâ.—Continue his diet.

Weather foggy and cloudy; wind NNW.

23d, three P.M.—Found him easier today, and lying on his back, but very much exhausted from the exertion of coughing and expectorating in the night, which commenced between the hours of three and four. He was almost suffocated by the orthopnea, but, after two severe efforts, threw up sixteen ounces of dark blood, but not coagulated as was the case at first; after which, till the time I saw him, he expectorated a pint of yellow phlegm, mixed with blood very dark in colour. Pulse quick and weak; bowels opened three times: the first evacuation was constipated. Has made a pint and a half of water, not so dark as before, which has precipitated the brickdust sediment. The pain is now confined to the eighth left rib, at its sternal extremity. Applied the stethoscope: the wheeze the same as yesterday, but not so violent.

Medicamenta repet^r.

Weather milder, but cloudy; wind W.

24th.—Rather easier. Threw up, during the night, seven ounces of grumous blood; the expectoration of phlegm not so profuse, but mixed still with clotted blood. Respiration easier.

Medicamenta repet^r.

Wind WNW.

From the 24th of December to the 2d of January, 1827, there was hardly any alteration; there was, perhaps, a shade for the better; but, on January 3d, he was attacked as on the 22d of December. I ordered him the same remedies. The stethoscope was applied, with the same results. Wind W.

January 4th.—The same as on the 23d December. Wind N. with snow.

5th.—Easier than yesterday in every respect.

He continues to get better and better every day, the morning paroxysm gradually lessening, until the 19th, when he again complained of great difficulty of breathing, with giddiness and nausea. He complains likewise of being unable to digest the most simple nutriment in a solid state, feeling heavy and fatigued after every slight meal. The stethoscope gave a more satisfactory result as to respiration. The mucous wheeze was still on the left side, unaccompanied by the gurgling in the axilla; on the right side, the sound was crepitating; but neither of these sounds could be distinguished below the seventh rib. Has no pain at the scrobiculus cordis. Wind E.

Ordered him a blister; the mixture and pill as before.

20th.—Had a paroxysm at the usual hour: threw up seven ounces of grumous blood. The phlegm is yellow and opaque, very viscid, mixed with clotted blood. Bowels opened ten times. Voided about two pints of urine, of a light straw colour, with a white sediment. Pulse quick and weak. Wind E.

Continue the mixture.

From this time until the 8th of February he continued mending, but today has the appearance of being threatened with another attack. Wind E.

Ordered him the usual medicines.

February 9th.—Had a paroxysm during the night, but only threw up phlegm, mixed with about one ounce of blood.

Repet^r medicamenta.

18th.—He has continued to gain ground until today, when the difficulty of breathing, &c. returned, but not to a great degree. Wind E.

Repeat the blisters, pills, and medicines.

19th.—Had a paroxysm at the usual hour during the night; expectorated only one ounce of grumous blood; after which he fell asleep, and was for five hours in a profuse perspiration. The phlegm now is very dense, and of a yellow colour; the quantity each day about ten ounces: water was added, but the mucus adhered so strongly to the bottom of the vessel that only two-thirds floated. His bowels have been moved seven times. Passes from a pint and a half to two pints of urine daily. His skin is generally moist; his pulse not so quick or weak; his breathing altogether better. Can lie a little on the left side, but, on turning, describes his sensation as of something falling like a substance, in the lower parts of the thorax, towards the side he lies upon: this feeling has been constant during the whole of his illness, so constant that he refers to it, himself, as to a kind of internal criterion of his own state of health. He considers that he has a cavity in a certain part of the thorax; of course, this falling originates from the consolidated lung.

Ordered him mutton-broth, and this mixture: ℞. Quin. Sulph. gr. iv.; Syr. Papav. ℥ss.; Acid. Sulph. dil. m. xxx.; Aq. pura ℥vj.; Magn. Sulph. ℥ss. M. capiat coch. iij. sextâ quâque horâ.

20th.—Found him much exhausted, the mixture having purged him excessively, yet better in every other respect. Not the least appearance of blood in the expectoration.

Ordered him the mixture without the salts.

March 16th.—Has continued to improve ever since. The expectoration is much lessened, as he only throws up daily three ounces of phlegm, which is transparent and colourless, floating in water, and not adhering as before to the vessel.

Continue his mixture.—Diet to be chicken or rabbit, whey, vegetables or fruit.

28th.—Is now as well as he has been for the last two years, but finds himself unable to digest with tolerable facility animal food.

Ordered to live on milk, vegetables, and fruits; to omit his medicines; and directed him to avoid all exposure to cold air or variable temperatures, especially to the easterly winds.

April 29th, 1827.—He says that he is better than he has been for the last two years, and that his present diet so well agrees with him that he never will be induced to change it. His bowels are open constantly; his urine passes more regularly, and in sufficient quantity; he has little of the cough, but when the wind is in the east; his respiration is good, unless he draws a very deep inspiration, and then he feels pain at the sternal extremity of the eighth rib on the left side. On applying the stethoscope, I found no respiratory murmur on the left side, but it commenced about the seventh rib on the right side. There is not the least sound communicated by percussion on the left side, but distinctly perceived on the right wherever the respiratory murmur was present; the resonance was then distinct.

There was neither pectoriloquy or œgophony throughout the whole of the complaint; but that there is hepatization of the lungs, from the eighth or ninth rib on both sides down to the extremities of the lobes, there can be no doubt. I intend to watch narrowly this patient: he has gone through great disease, and must expect another attack. Should he sink under it, I will beg a post-mortem examination, and clear all doubts with regard to the actual causes of, and absolute seat of his disorder.

11, *Park-street, Grosvenor-square;*
*March* 1828.

